# Stakeholder engagement to co-design implementation strategies for integrating depression management into HIV care services in Senegal

**DOI:** 10.1186/s43058-025-00801-1

**Published:** 2025-11-17

**Authors:** Charlotte Bernard, Keitly Mensah, Kathryn L. Lovero, Hawa Abou Lam, Hélène Font, Judicaël Malick Tine, Salaheddine Ziadeh, Ibrahima Ndiaye, Awa Diagne, Maguatte Ndiaye, Jean Augustin Diégane Tine, Antoine Jaquet, Ndeye Fatou Ngom, Moussa Seydi, the IeDEA West Africa Cohort Collaboration (Group), Charlotte Bernard, Antoine Jaquet, Moussa Seydi, Armel Poda, Bobo Dioulasso, Oliver Ezechi, Eugene Messou, Henri Chenal, Kla Albert Minga, Aristophane Tanon, Ephrem Mensah, Caroline Yonaba, Lehila Bagnan Tossa, Jocelyn Dame, Sylvie Marie N’Gbeche, Kouadio Kouakou, Madeleine Amorissani Folquet, François Tanoh Eboua, Fatoumata Dicko Traore, Agatha David, Elom Takassi, Didier Koumavi Ekouevi, François Dabis, Renaud Becquet, Karen Malateste, Olivier Marcy, Marie Kerbie Plaisy, Elodie Rabourdin, Thierry Tiendrebeogo, Désiré Dahourou, Sophie Desmonde, Julie Jesson, Valeriane Leroy, Raoul Moh, Jean-Claude Azani, Jean Jacques Koffi, Eric Komena, Maika Bengali, Abdoulaye Cissé, Guy Gnepa, Apollinaire Horo, Simon Boni, Eulalie Kangah, Corinne Moh, Jeanne Eliam, Ighovwerha Ofotokun, Chris Martin, Noelle Benzekri, Geoffrey Goettlieb, Olivia Keiser

**Affiliations:** 1 University of Bordeaux, National Institute for Health and Medical Research (INSERM) UMR 1219, Research Institute for Sustainable Development (IRD) EMR 271, Bordeaux Population Health Centre, 146 Rue Léo Saignat, 33076 Bordeaux, Cedex France; 2 Infection, Antimicrobials, Modelling, Evolution (IAME), UMR 1137, Université Paris Cité, InsermParis, France; 3 Department of Sociomedical Sciences, Columbia University Mailman School of Public Health, New York, NY USA; 4 Laboratoire de Sociologie, Anthropologie Et Psychologie Sociale (LASAP), ETHOS, Université Cheikh Anta Diop, Dakar, Sénégal; 5 Service Des Maladies Infectieuses Et Tropicales, CHNU de Fann, Dakar, Senegal; 6 Global Mental Health Lab, Teachers College, Columbia University, New York, NY USA; 7 Faculté de Santé Publique, Université Libanaise, Sidon, Lebanon; 8 Service de Psychiatrie, CHNU de Fann, Dakar, Senegal; 9 Centre National de Lutte Contre Le VIH/SIDA/Hépatites, Dakar, Senegal; 10 Division Nationale de Lutte Contre Le VIH/SIDA, Dakar, Senegal; 11 Division Santé Mentale, Ministère de La Santé Et de L’action Sociale, Dakar, Senegal; 12 Centre de Traitement Ambulatoire, CHNU de Fann, Dakar, Senegal; 13 Département de Médecine de L, Université Alioune Diop de Bambey, Bambey, Senegal

**Keywords:** Depression, HIV infection, Sub-Saharan Africa, Implementations Strategies, Implementation Mapping

## Abstract

**Background:**

Depression is highly prevalent in people living with HIV (PWH), affecting their daily life and HIV outcomes. Following a successful pilot study to treat depression in PWH with Group Interpersonal Therapy, we examined its implementation potential. Despite a strong willingness for its adoption routine practice, formal integration of mental health services into HIV care remained challenging. Using Implementation Mapping, we aimed to select and specify a set of implementation strategies to integrate depression services into Senegalese HIV care.

**Methods:**

For each step of depression services (i.e. screening, diagnostic confirmation/referral, and treatment), we selected potential implementation strategies using the Expert Recommendations for Implementing Change (ERIC). During a 3-day workshop, 14 different stakeholders, including doctors, social workers, community health workers, a psychiatrist, a socio-anthropologist and local health officials, reviewed and discussed strategies selected for each implementation step. Each participant also voted on the importance and feasibility of each strategy, using a Likert scale from 1 to 5 (5 = very high importance or feasibility). Scores were then plotted on a 'go-zone' graph. Details of strategies ranked as important and feasible were then specified by stakeholders**.**

**Results:**

Forty-eight strategies were identified. Among them, 62,5% were considered as highly important and feasible, 31,3% as important but with concerns about feasibility, 6,2% as not very important or feasible. A total of 46 distinct implementation strategies, derived from 21 ERIC strategies and corresponding to 8 ERIC thematic clusters, were selected for the final implementation plan. Materials needed to implement and monitor implementation (i.e. registers, decision tree, patient’s record) were validated during the workshop. Finally, a summary of the implementation plan for integrating depression management into HIV care services in Senegal was elaborated.

**Conclusions:**

A systematic approach was used to collaboratively develop an implementation plan to integrate depression management into HIV care in Senegal. Informed by various stakeholders, this work can facilitate a national dissemination of the integration program and may offer a useful reference for developing similar programs for PWH in other settings.

**Supplementary Information:**

The online version contains supplementary material available at 10.1186/s43058-025-00801-1.

Contributions to the literature

- This study is the first to report an implementation plan to integrate depression care in HIV services in Senegal, based on stakeholders’ engagement.
- This study reported the process to select and co-design implementation strategies, related to each step of depression services (i.e. screening, diagnostic confirmation/case referral, treatment). Stakeholders involved in the co-design process were medical doctors involved in HIV care, a psychiatrist, social workers, a community health worker, a social anthropologist, a representative of the National AIDS Council, and a representative of the Mental Health Division.
- This study clarifies how use of Implementation Mapping through stakeholder engagement can produce an implementation plan for depression care integration.
- This approach provides a set of strategies for integrating depression management into HIV services across the country and could serve as a guide for developing similar programs in other low-resource contexts.

## Introduction

Senegal is a francophone West African country with limited resources and a population of over 16 million as of 2020 [1]. Despite an increase of 14% compared to 2019, only 43 psychiatrists were providing services in 2020 [1] resulting in a psychiatrist-to-population ratio of approximately 0.3 per 100,000 inhabitants—significantly lower than in high-income countries (i.e. in the OCDE, 15.6 psychiatrists per 100,000 inhabitants [2]). As in many other sub-Saharan African (SSA) countries, healthcare provision is geographically unbalanced, typically concentrated in the capital [3], although half the population live in rural areas. The need to improve access to care and to develop partnerships with other national programs, such as the HIV/AIDS program, to integrate services has been highlighted in the recent National Strategic Plan of the Senegalese Mental Health Division 2024–2028 [1].

Mental health conditions negatively impact engagement in the HIV care continuum [4] and may compromise UNAIDS 2030 targets [5]. The most common psychiatric disorder in people living with HIV (PWH) is depression and its prevalence is high in SSA countries (14 to 32%) [6]. In addition to its functional, social, and professional consequences, depression also affects HIV outcomes, including adherence to antiretroviral treatment (ART) [7–9]. A meta-analysis reported that PWH with depressive symptoms had 42% lower ART adherence than PLHW without any depressive symptoms [10]. In Senegal, around 0.3% of the population aged 15–49 years old were living with HIV in 2022 [11]. Although data on depression prevalence in PWH in Senegal are limited, one study estimated it at 18% [12]. Another study reported a similar prevalence specifically in PWH aged 50 years and older [13]. Several calls for action to integrate mental health into HIV care have been made [4, 14].

In response to this need, we conducted a pilot implementation of group Interpersonal Therapy (IPT), based on a task-shifting approach, in four HIV care clinics in Dakar, the capital of Senegal [15–17]. IPT’s treatment principles and the rationale for its use have been described in detail elsewhere [18, 19], including its demonstrated effectiveness in the treatment of depression as shown in systematic reviews [20, 21]. Group IPT is recommended by the World Health Organization (WHO) for the treatment of depression in Low- and Middle-Income Countries (LMIC). Task shifting is also widely recognized as an effective method for delivering psychological interventions [22, 23]. Group IPT was first used in SSA to treat depression in the general population in Uganda [24] and then in PWH [25–28]. In our pilot study, we observed high acceptability, feasibility, and benefits of group IPT to treat depression as well as improve the social and professional lives of PWH [29, 30]. Increased clinical mental health skills were also reported among professionals involved in the project [30]. Our successful experience led us to extend the program to two primary and secondary level health facilities outside Dakar, with positive results confirming our previous findings [31]. Replicability aside, one implication of the study was the recognition that formal integration of mental health services into HIV care was critical to the sustainability and scale-up of group IPT.

Formal integration of depression care encompasses multiple steps such as screening, diagnosis confirmation, case referral, and treatment. Multi-level barriers and facilitators can hinder or foster the successful implementation of depression care, some of which have been previously identified and reported in Senegal [30–33]. Thus, to promote the integration of depression treatment services into routine HIV care, systematic approaches to developing contextually-appropriate strategies for implementation are needed [34–36].

Implementation Mapping is a five-step systematic process for selecting adequate strategies needed to overcome barriers to implementation and promote the adoption, application, and sustainability of evidence-based interventions in real-world settings [37]. Based on insights from both the implementation science field and Intervention Mapping research, this method guides the development of implementation strategies in a systematic and replicable way [37, 38]. This method is also useful for engaging stakeholders in the process, following a structured approach. In particular, it has been successfully used to design implementation strategies for mental health services in Mozambique, involving multilevel stakeholders [39].

Using the core concepts of Implementation Mapping, this study aimed to select and co-design with various stakeholders a set of implementation strategies to integrate depression services into Senegalese HIV care.

## Methods

### Implementation mapping

Implementation Mapping consists of five iterative tasks [37]: 1) assessment of implementation needs, after identifying program adopters and implementers; 2) identification of implementation outcomes, performances objectives and determinants of change; 3) selection of implementation strategies; 4) creation of an implementation protocol; and 5) evaluation of implementation outcomes and plan. We used Tasks 1–4 to prepare, build and conduct a joint workshop with stakeholders aimed at developing together implementation strategies by co-designing them. Tasks 1 to 4 have been adapted to our study (Figure S1).

### Preparatory stages for selecting implementation

To prepare for the selection and co-design of suitable strategies, we used Tasks 1 and 2 to understand needs, assets and identify expected implementation outcomes, indicators and determinants of change.

First, implementation planners identified adopters and implementers. Implementation planners were Senegalese, French, and American experts in public health (*N* = 2), infectious diseases (*N* = 3), socio-anthropology (*N* = 1), implementation science (*N* = 2) and mental health (*N* = 3) as well as three representatives of Senegalese national policy makers (National AIDS Council, AIDS Control Division and Mental Health Division of the Ministry of Health and Social Action). The adopters consisted of four site directors (representing every clinic where group IPT had previously been implemented), the National AIDS Council, the AIDS Control Division and the Mental Health Division of the Ministry of Health and Social Action. Implementers were trained staff involved in the different steps of integration (i.e. screening, diagnosis and case referral, treatment) (4 per site), including: doctors, nurses, social workers, community health workers, secretaries, and pharmacists working in HIV services.

Second, we identified facilitators and barriers to the implementation of systematic screening and group IPT in HIV services in Senegal. This step was conducted in different HIV care contexts in Senegal prior to the present study, using qualitative interviews with patients and healthcare professionals analyzed thematically. Related work has been published as original papers [30–32]. In the present study, these determinants were synthesized to inform selection and specification of implementation strategies.

Third, implementation outcomes were selected based on our previous work and guided by implementation science frameworks (in particular Proctor’s Implementation Outcomes Framework, Bowen’s feasibility Framework, Purcell definitions of acceptability and the RE-AIM framework), to assess acceptability, feasibility, adoption, fidelity, penetration and sustainability [40–44]. The underlying mechanisms of change related to identified needs and proposed implementation strategies were also identified using a widely recognized taxonomy (i.e. Kok et al. taxonomy) [45].

Reflection on the integration of depression treatment services into HIV care focused on 4 main steps: general implementation process, depression screening, diagnosis confirmation / case referral, and treatment. An iterative process was required to optimize the final implementation plan.

### Selection of implementation strategies

Prior to the workshop and based on results of Implementation Mapping Tasks 1 and 2, the implementation planners selected potential implementation strategies by reviewing the Expert Recommendations for Implementing Change (ERIC) [46]. The ERIC reports a compilation of implementation strategies refined by a group of experts to standardize reporting and evaluation [46]. The selected strategies were tailored to the context and the objectives of the implementation. The ERIC thematic clusters were used to classify the selected strategies [47]. For each step, we created a simplified logic model [48], using Miro software (https://miro.com/), to link barriers/facilitators and needs to the selected strategies and outcomes.

To engage stakeholders in the selection and the co-design of implementation strategies, we then organized a 3-day workshop in August 2024. For each step: i) general implementation process, ii) screening, iii) diagnosis confirmation / case referral, and iv) treatment, implementers and adopters were invited to review and discuss the selected strategies, related actions and other aspects of their specifications [49].

Workshop participants included four medical doctors involved in HIV care, a psychiatrist, four social workers, a community health worker, a social anthropologist, a representative of the National AIDS Council, and a representative of the Mental Health Division. All participants, except the representative of the national institutions, were involved in the previous Senegalese group IPT projects [29–32]. They had various and complementary experiences: lived experiences from professionals—coming from different types of health facilities—that informed both the design and perceived feasibility and importance of the proposed strategies and experience in building implementation plans from representative of the national institutions. The workshop was facilitated by a mental health researcher (CB), the scientific coordinator of the previous works. All workshop sessions included a general presentation by the mental health researcher and interactive discussions. The Wooclap software (https://www.wooclap.com/fr/) was used to facilitate the sessions, allowing live but anonymous polling and voting on strategies and strategy components. Rates were not visible during the vote.

During Workshop Day 1, the lead author (CB) presented an overview of the workshop objectives, the general context of mental health in PWH and a summary of the different research conducted on the topic in Senegal. The rest of the day was focused on developing strategies related to the screening step. Workshop Day 2 was focused on diagnosis confirmation and case referral (first part of the day) and treatment (second part of the day) steps. Workshop Day 3 was focused on the general implementation process. For each step, CB presented a simplified logic model presenting the facilitators and barriers observed in previous work, as well as identified needs based on those observations, and expected outcomes. Then, step by step, addressing each need, pre-selected strategies were presented. For each strategy, we discussed the formulation and the required action (i.e., relevant, relevant but modifications needed, or irrelevant) associated therewith. This allowed us to discuss in detail how to implement each strategy. When participants had disagreements, an open dialogue was initiated by CB who led the workshop to understand the reasons behind them. Consensus between participants was always reached before finalizing a decision. Each participant also voted on the importance and feasibility of each strategy, using a Likert scale from 1 to 5 (5 = very high importance or feasibility). Participants scores were averaged to create an overall importance or feasibility scores. The importance and feasibility scores for each strategy were plotted on a 'go-zone' graph [47], using a threshold of 4.5 for each dimension. This threshold was chosen to avoid a ceiling effect. The resulting graph was divided into four quadrants: Q1 represents strategies with high importance and feasibility; Q2 high importance but mixed feasibility; Q3 high feasibility but low importance; Q4 low importance and feasibility. Finally, following Proctor’s recommendations for specifying implementation strategies [49], we specified during the workshop each strategy selected for inclusion in the final implementation plan, including a description of the actor (who leads the strategy), action required, action target (who is affected by the action), temporality (when the strategy is used), dose (how often the action needs to be done), and the affected outcomes. For the justification of each strategy and each step of this process, the reader is referred to the logic models (Supplementary data, Figures S2 to S5). During the workshop, open discussions welcomed all comments, leading to emergent themes and suggestions from participants that were incorporated into the final analysis.

### Ethical consideration

Oral consent was obtained for all workshop participants. No individual data were collected that required written consent. This research is part of the project 'intervention depression' knowledge transfert which received ethical approval (protocol-number: SEN22/49; approved-number: 0000102/MSAS/CNERS/SP).

## Results

**Fig. 1:**
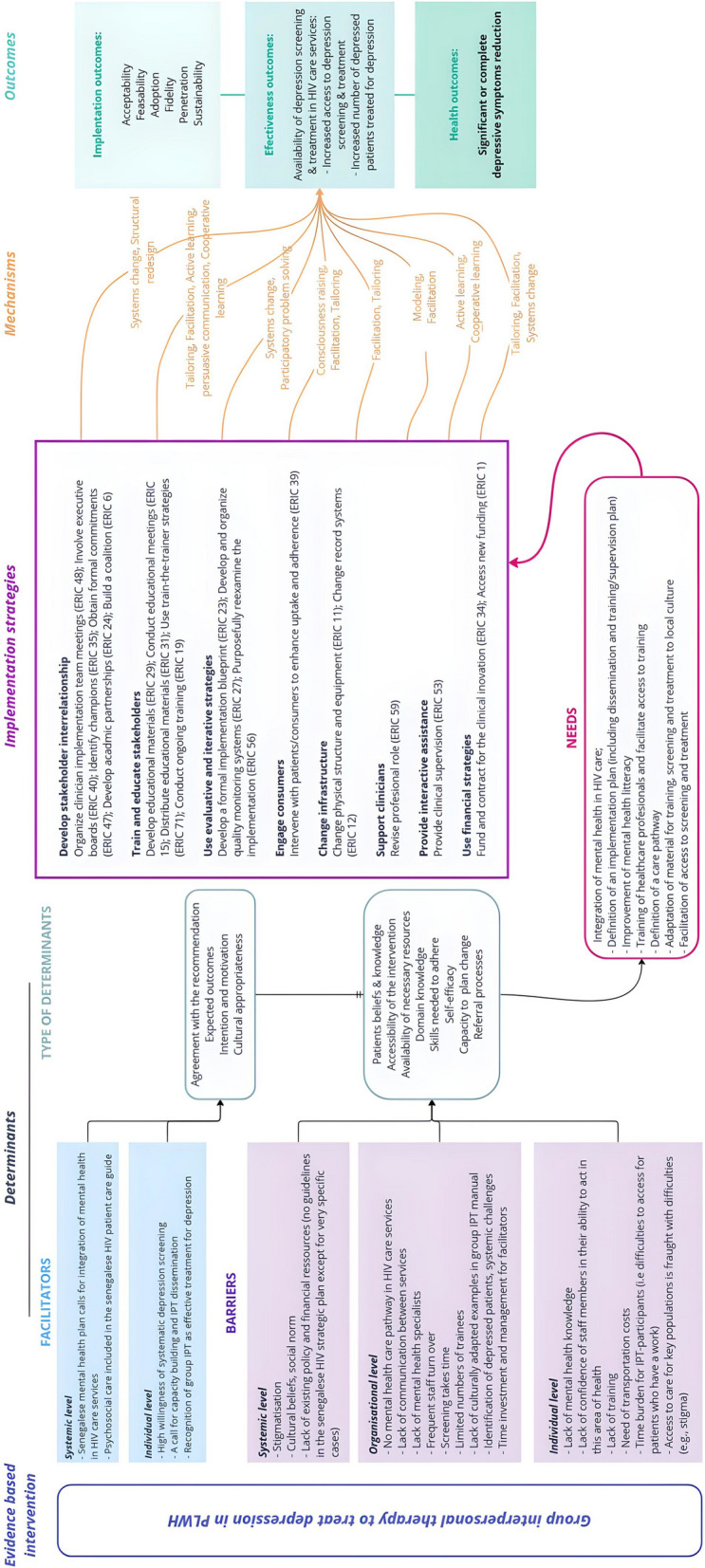
Logic model for integrating depression management into HIV care in Senegal, guided by intervention mapping. Implementation strategies were identified based on the Expert Recommendations for Implementing Change (ERIC) [46] and were classified using ERIC clusters [47]

The global implementation logic model we developed for integrating depression care into HIV services, guided by Implementation Mapping, is presented in Fig. 1.

### Implementation needs

Based on previous works on depression screening and group IPT implementation conducted in different contexts in Senegal [29–32], we observed a high willingness to integrate depression management into HIV clinics. At the individual level, confidentiality and patient privacy as well as the lack of knowledge about mental health were the main barriers. At the organizational level, the major concern was time demands. At the systemic level, cultural beliefs, social norms and stigmatization were reported as the main barriers. We also identified related needs that require to be addressed to facilitate implementation and sustainability: 1/ training healthcare professionals (individual level); 2/ definition of a care pathway in each clinic, including an available trusted staff and adapted tools (organizational level); 3/ improvement of mental health literacy in communities (systemic level); and 4/ integration of mental health into national HIV care guidelines (systemic level). Specific needs were reported for group IPT: reimbursement of transport costs to facilitate access to care and ensure access to specific populations (i.e. key population, workers). Barriers, facilitators and needs are summarized in Fig. 1 and detailed for each step in Supplementary data (Figures S2 to S5). Implementation outcomes and performance targets were selected based on our previous works but were completed specifically regarding screening step, adoption (especially for scaling up) and sustainability. They are presented in Table S1. Main outcomes were validated orally by workshop participants.

### Implementation strategy selection

We formulated and presented to the stakeholders 48 strategies to address implementation needs: 15 for the general implementation process, 13 for the screening step, 8 for the diagnosis confirmation and case referral step, and 12 for the treatment step. For each step, we included both implementation and monitoring strategies: 12/15 and 3/15 for the general implementation process, respectively; 10/13 and 3/13 for screening step; 6/8 and 2/8 for diagnosis confirmation and case referral step; 9/12 and 3/12 for treatment step. We discussed strategies one by one and we detailed the actions formulated for each strategy to the workshop participants. We also discussed other aspects of specification, particularly dose, whenever necessary.

Table 1 presents each selected strategy and the relevance, importance and feasibility of each of them according to the workshop’s participants. The strategies have been numbered and this number is used in the text to facilitate reading. In Tables 2 and 3, we presented the strategies included in the final implementation plan and their specification for each step. Between Tables, reformulations or clarifications of strategy components were made based on participant consensus. All the discussions that took place during the workshop are summarized below.

**Table 1:** Selected implementation strategies: relevance, importance, feasibility for integrating depression management into HIV services in Senegal

**ERIC clusters**	**N°**	Implementation strategy*	**Relevant formulation (%)**			**Importance**^§^	**Feasibility**	**Go-Zone**^**a**^
			**Yes**	**Modifications requested**	**No**			
**General implementation process**								
**Develop stakeholder interrelationships**	1	Create a depression care team including implementers and adopters at settings	100	0	0	5	4.6	Q1
	2	Collaborate with the depression care team to create intervention flowchart	73	27	0	4.6	4.6	Q1
	3	Share implementation/dissemination plan with national and local policymakers	83	17	0	4.9	4.6	Q1
	4	Identify person at setting to serve as depression care team leader	91	9	0	5	4.2	Q2
	5	Obtain approval and commitment from directors of settings	100	0	0	5	4.8	Q1
	6	Create partnership with local institutions (national school of social workers, universities)	50	50	0	4.3	3.9	Q4
	7	Create a national working group involving different stakeholders	100	0	0	5	5	Q1
	8	Establish a national directory of psychiatrists to manage active suicidal risks	100	0	0	5	5	Q1
**Train and educate stakeholders**	9	Train local psychiatrists to group Interpersonal Therapy (IPT) or Interpersonal Counselling (IPC)	100	0	0	4.9	4.6	Q1
	10	Establish a dynamic training plan based on train the trainers’ strategies	100	0	0	5	4.8	Q1
**Use evaluative and iterative strategies**	11	Create detailed implementation plan	100	0	0	5	4.6	Q1
	12	Maintain continuous communication between implementation planners and team lead	92	8	0	4.8	4.1	Q2
	13	Organize meetings with implementation planners and intervention team	100	0	0	4.8	4.3	Q2
	14	Monitor progress	92	8	0	4.9	4.5	Q1
**Utilize financial strategies**	15	Define a budget allocated to this dissemination and identify resources	92	8	0	4.9	4.3	Q2
**Screening step**								
**Engage consumers**	16	Educate patients about depression	50	50	0	4.6	4	Q2
**Train and educate stakeholders**	17	Develop specific and adapted training on depression	75	25	0	4.8	4.7	Q1
	18	Use non-stigmatizing, adapted and appropriated language to introduce and do screening	88	12	0	4.8	4.8	Q1
	19	Train staff about depression, symptoms and the screening tool	75	25	0	4.9	4.8	Q1
	20	Distribute support materials for screening	85	15	0	4.8	4.8	Q1
	21	Identify clinicians or experimented trained social workers to train others in the screening step	85	15	0	4.7	4.6	Q1
	22	Conduct refreshment training for screening	44	11	44	4.6	4.5	Q1
**Change infrastructure**	23	Develop an adapted screening tool	100	0	0	4.9	4.9	Q1
	24	Use a digital tool for screening	73	27	0	4.7	4.1	Q2
	25	Identify adequate space for screening	100	0	0	4.9	4.7	Q1
	26	Create a screening record	100	0	0	4.9	4.8	Q1
**Support clinicians**	27	Identify who does the screening	82	18	0	5	5	Q1
**Provide interactive assistance**	28	Supervise screening	100	0	0	5	4.6	Q1
**Diagnosis confirmation and case referral step**								
**Engage consumers**	29	Bring patients with positive screening directly to physician in charge for confirmation	22	67	11	4.4	4.1	Q4
	30	Educate patients about depression treatment after confirmation	100	0	0	5	4.7	Q1
	31	Provide initial IPT session on day of screening	17	83	0	4.6	3.6	Q2
**Train and educate stakeholders**	32	Use non-stigmatizing language to give feedback on confirmation	100	0	0	4.7	4.8	Q1
	33	Conduct refresher training for confirmation	27	9	64	3.6	3.4	Q4
**Change infrastructure**	34	Create and use a decision tree for patient monitoring	100	0	0	4.5	4.3	Q2
	35	Use of an adapted and digital tool to confirm depression	20	80	0	4.6	3.8	Q2
**Provide interactive assistance**	36	Supervise confirmation step	0	100	0	4.7	4.2	Q2
**Treatment step**								
**Engage consumers**	37	Call patients on day prior to group IPT sessions	91	1	0	5	4.2	Q2
	38	Propose alternative treatment in specific cases	83	17	0	4.8	3.8	Q2
**Train and educate stakeholders**	39	Use of context-sensitive examples in training materials	100	0	0	4.9	4.7	Q1
	40	Train social workers to Interpersonal Counselling (IPC)	33	67	0	4.8	3.9	Q2
	41	Conduct refresher training for group IPT	100	0	0	4.9	4.7	Q1
**Change infrastructure**	42	Identify adequate space for group IPT sessions	91	9	0	5	4.6	Q1
	43	Use specific IPT record for each patient	100	0	0	4.9	4.6	Q1
	44	Use a IPT summary form to monitor treatment	100	0	0	4.9	4.6	Q1
**Support clinicians**	45	Train at least 2 providers in each setting	100	0	0	4.9	4.6	Q1
	46	Reorganize working hours to integrate therapy in facilitators' daily working activities	45	55	0	4.6	4.3	Q2
**Provide interactive assistance**	47	Supervise group IPT	92	8	0	5	4.8	Q1
**Utilize financial strategies**	48	Identify a source of funding to help patients to come to therapy (transportation costs)	100	0	0	4.9	3.5	Q2

^*^Monitoring strategies are highlighted in green

^§^Each participant voted on the importance and feasibility of each strategy, using a Likert scale from 1 to 5 (5 = very high importance or feasibility). Participants scores were averaged to create an overall importance or feasibility scores

^**a**^Results obtained from the go-zone plot: Q1 represents strategies rated with high importance and feasibility; Q2 represents strategies rated with high importance but mitigate feasibility; Q3 represents strategies rated with high feasibility but low importance; Q4 represents strategies rated with low importance and feasibility

**Table 2:** General Implementation and Screening steps: strategies specifications for integrated depression services in Senegalese HIV care

Implementation strategy*		Actor	Action	Target	Temp	Dose	Outcomes affected
**General implementation process**							
**Develop stakeholder interrelationships**							
o Create a depression care team including implementers and adopters at settings (ERIC 48)		IP	Identify staff who will be involved in the integration of depression care within the clinic	A,I	Prep	Once	Adoption, sustainability
o Collaborate with the depression care team to create intervention flowchart (ERIC 48)		IP	Discuss with adopters and implementers about the most suitable care pathway adapted to the functioning of their service (who does what, where, when, etc.)	A,I	Prep	Once	Acceptability, Adoption, sustainability
o Share implementation/dissemination plan with national and local policymakers (ERIC 40)		IP	Present and deliver copies of implementation plan of Ministry of health and local health departments	A	Prep	Once	Adoption, sustainability
o Create a national working group to elaborate the implementation plan (ERIC 40)		IP	Create a national working group to elaborate the implementation plan and involving different stakeholders	A	Prep	Once	Adoption, sustainability
o Identify persons at setting to serve as depression care team leaders (ERIC 35)		IP,A	Discuss with adopters to identify one implementer that can coordinate activities in each setting and help for monitoring activities, with specific characteristics of leadership and self-motivation	I	Prep	Once	Adoption, sustainability
o Obtain approval and commitment from directors of settings (ERIC 47)		IP	Present and obtain authorization for the implementation plan from the administration of each facility	A	Prep	Once	Adoption, sustainability
o Create parternship with local institutions (national school of social workers, universities) (ERIC 24)		IP	Integrate intervention training into existing training curricula to increase the number of trainees and limit costs	A	Prep	Once	Adoption, sustainability
o Establish a national directory of psychiatrists (ERIC 6)		IP	Establish a national directory of psychiatrists to manage active suicide risks	I	Prep	Once	Feasibility, adoption, sustainability
**Train and educate stakeholders**							
o Train local psychiatrists or psychologists in group IPT and IPC (ERIC 19)		IP	Train local mental health specialists in group IPT or IPC to create a workgroup to facilitate dissemination, autonomy and commitment	A,I	Prep	Once	Adoption, sustainability
o Establish a dynamic training plan based on train the trainers’ strategies (ERIC 71)		IP	Establish a training plan that includes the progressive training of facilitators into supervisors to gain autonomy. It will include the confirmation step, group IPT/IPC	A	Prep	Once	Adoption, feasibility, sustainability
**Use evaluative and iterative strategies**							
o Create detailed implementation plan (ERIC 23)		IP	Develop a detailed plan explaining objectives, roles, activities, timeline, budget and expected outcomes	A, I	Prep	Once	Adoption, sustainability
o Maintain continuous communication between implementation planners and team lead (ERIC 27)		IP	Garantee open comunication between IP and intervention team to resolve urgent issues	I	Imp	Cont	Fidelity, penetration, retention
o Organize meetings with IP and intervention team (ERIC 27)		IP	Report outcomes and hold open discussion on implementation and emerging barriers	I	Imp	Ev. 3 months	Fidelity, acceptability, penetration
o Monitor and evaluate progress (ERIC 56)		IP	Monitor and evaluate the dissemination of the intervention in new settings	A,I	Imp	2x/year	Adoption, sustainability, fidelity, acceptability, penetration
**Utilize financial strategies**							
o Define a budget allocated to this dissemination and identify ressources (ERIC 34)		IP	Identity and quantify the main costs required for training and therapy implementation/maintenance to allocate a budget and/or find sources of fundings	A	Prep	Once	Adoption, sustainability
**Screening step**							
**Engage consumers**							
o Educate patients about depression (ERIC 39)	I		Educate patients with adapted tools, about symptoms of depression, how to identify them and the importance of help seeking	P	Imp	Cont	Acceptability, penetration
**Train and educate stakeholders**							
o Develop specific and adapted training on depression (ERIC 29)	IP		Develop co-constructed and friendly training materials created specifically, adapted to social norms and beliefs and including local examples + that can be used by existing staff to train new staff	P	Prep	Once	Acceptability, Fidelity, Sustanability
o Use non-stigmatizing, adapted and appropriated language to introduce and do screening (ERIC 29)	I		Use clear, simple, appropriate language to describe screening and explain positive screening	P	Imp	Cont	Acceptability, Penetration
o Train staff about depression, symptoms and the screening tool (ERIC 15)	IP		Train staff about depression, how to conduct screening using a specific tool and specific skills to increase active listening and empathy	I	Prep	Once + Sup	Acceptability, Fidelity, Sustanability
o Distribute support materials for screening (ERIC 31)	IP		Create technical brochure explaining depression symptoms, how to manage suicidal ideations and how to screen	I	Imp	Once	Fidelity, Penetration
o Identify clinicians or experimented trained social workers to train others in the screening step (ERIC 71)	IP, A		Work with head of services to identify one implementer that can train new staff when necessary	I	Prep	Once	Feasability, Fidelity, Sustainability
**Change infrastructure**							
o Develop an adapted screening tool (ERIC 11)	IP		Adapt the screening tool to facilitate its administration and translate it in local language	I	Prep	Once	Acceptability, Adoption, Feasibility,
o Use a digital tool for screening (ERIC 11)	IP		Create and use a digital tool that auto-calculates scores to facilitate screening	I	Prep	Once	Acceptability, Adoption, Feasibility, Sustainability
o Identify adequate space for screening (ERIC 11)	I		Find quiet and private space to conduct screening	P	Imp	Once	Acceptability,
o Create a screening record (ERIC 12)	IP		Set up a register including the number of patients screened and the number referred for confirmation, reviewed by the intervention team once a month	I	Prep	Once, Cont. Use	Feasibility
**Support clinicians**							
o Identify who does the screening (ERIC 59)	A, I		Decide with adopters and implementers who can do screening in the HIV care service (adaption to the context)	P	Prep	Once	Adoption, Feasibility, Sustainability
**Provide interactive assistance**							
o Supervise screening (ERIC 53)	IP		Supervise screening after didactic training regularly during the first week, at week 3, month 3 and every 6 months	I	Imp	W1&3, M3, ev.M6	Fidelity, Sustainability

^*^Monitoring strategies are underlined in green

*Abbreviations: A* adopter, *Cont.* Continuous, *Ev* every, *I* implementers, *Impl* Implementation, *IP* implementation planners, *Prep* preparation, *Temp* Temporality, *M* Month, *W* week

**Table 3:** Depression diagnosis confirmation/case referral and treatment steps: Strategies specifications for integrated depression services in Senegalese HIV care

Implementation strategy	Actor	Action	Target	Temp	Dose	Outcomes affected
**Depression Diagnosis confirmation and case referral step**						
**Engage consumers**						
o Confirm depression diagnosis the same day as screening for patients with positive screening (ERIC 39)	I	Create a sheet form the patient will give to the physician during its consultation to confirm depression diagnosis. For specific cases (i.e. patients who collect their medication without a consultation), priority access to the consultation would be applied	I,P	Impl	Cont	Penetration
o Educate patients about depression treatment after confirmation (ERIC 39)	I	Provide psycho-education to stress the importance of treating depression and give an overview of group IPT	P	Impl	Cont	Penetration
o Provide initial IPT session shortly after screening (ERIC 39)	I	Organize with the trained social worker a session just after confirmation of depression or during the week after	P	Impl	Cont	Feasibility, Penetration
**Train and educate stakeholders**						
o Use non-stigmatizing and clear language to give feedback on confirmation (ERIC 29)	I	Use simple, clear and adapted terms to explain what is depression, its treatment and normalize depression	P	Impl	Cont	Acceptability, Penetration
**Change infrastructure**						
o Create and use a decision tree for patient monitoring (ERIC 11)	IP	Create and use a decision tree to define when a patient should be screened again, based on his or her screening tool score	I	Impl	Cont	Fidelity, Penetration
o Use of an adapted tool to confirm depression (ERIC 11)	IP	Use the MHGAP depression module on the application (on sheet form depending on the clinic context) to facilitate the confirmation	I	Impl	Cont	Acceptability, Adoption, Feasibility, Sustainability
**Provide interactive assistance**						
o Supervise confirmation step (ERIC 53)	IP	Supervise confirmation after didactic training regularly during the first month, then at month 3 and every 6 months	I	Impl	M1, M3, every M6	Fidelity, Sustainability
**Treatment step**						
**Engage consumers**						
o Call patients on day prior to group IPT sessions (ERIC 39)	I	Contact patients to remind them of upcoming session	P	Imp	Cont	Feasibility
o Propose alternative treatment in specific cases (ERIC 39)	I	Propose alternative to group treatment if the patients cannot come at the chosen time for the weekly sessions for very specific reasons (work or because of stigma (key population) and only if the patients have moderate symptoms	P	Imp	Cont	Feasbility, Penetration
**Train and educate stakeholders**						
o Establish a training plan (ERIC 71)	IP	Train facilitators to supervise new facilitators	I	Imp	Once	Feasibility, Sustainability
o Use of context-sensitive examples in training materials (ERIC 31)	IP	Integrate stories of patients who benefitted from group IPT as examples during training and in an adapted version of the WHO manual	I	Imp	Once	Adoption, Fidelity, Penetration
o Train social workers to Interpersonal Counselling (IPC) (ERIC 15)	IP	Train teams in interpersonal counselling to manage patients who are unable to attend the group and have moderate symptoms	I	Imp	Once	Acceptability, Feasibility, Adoption, Sustainability
o Conduct refresher training for group IPT (ERIC 19)	IP	Organize refresher training for group IPT to ensure fidelity	I	Imp	1 to 2 × per year	Fidelity
**Change infrastructure**						
o Identify adequate space for group IPT sessions (ERIC 11)	I	Find quiet and private space to conduct screening	P	Imp	Cont	Acceptability, Feasibility
o Use specific IPT record for each patient (ERIC 11)	I	Use specific IPT record for each patient, including personal inventory, IPT areas, in-between tasks, progression, etc., to facilitate group sessions	P	Imp	Cont	Adoption, Fidelity, Feasibility
o Use a IPT summary form to monitor treatment (ERIC 11)	IP	Use the IPT summary form to document symptoms evolution, absences, drop out, etc., reviewed by the intervention team leader at the end of the 8 IPT sessions for each group	I	Imp	Cont	Acceptability, Feasibility
**Support clinicians**						
o Train at least 2 providers in each setting (ERIC 59)	IP	Train a sufficient number of people to lighten the workload and facilitate access to treatment	I	Imp	Cont	Acceptability, Fidelity, Sustainability
o Think of the possibility to reorganize working hours to integrate therapy in facilitators' daily working activities (ERIC 59)	IP	Discuss on a case-by-case basis the possibility of reorganizing daily work activities to allow social workers to lead group sessions	A	Imp	Once	Acceptability, Feasibility, Adoption, Sustainability
**Provide interactive assistance**						
o Supervise group IPT (ERIC 53)	IP	Organize weekly supervision sessions for the 3 first facilitated IPT groups, then 2–3 times for each new group	I	Imp	Ev. week then 3 times per group	Fidelity, Sustainability
**Utilize financial strategies**						
o Identify a source of funding to help patients to come to therapy (transportation costs) (ERIC 1)	IP	Obtain a specific budget from authorities or find donors or to cover patient transport costs (depending on distance between home and place of care)	P	Imp	Cont	Feasibility, Penetration

Monitoring strategies are underlined in green

*Abbreviations: A* adopter, *Cont.* Continuous, *Ev* every, *I* implementers, *Impl* Implementation, *IP* implementation planners, *Prep* preparation, *Temp* Temporality, *M* Month, *W* week

### Implementation process

**Fig. 2:**
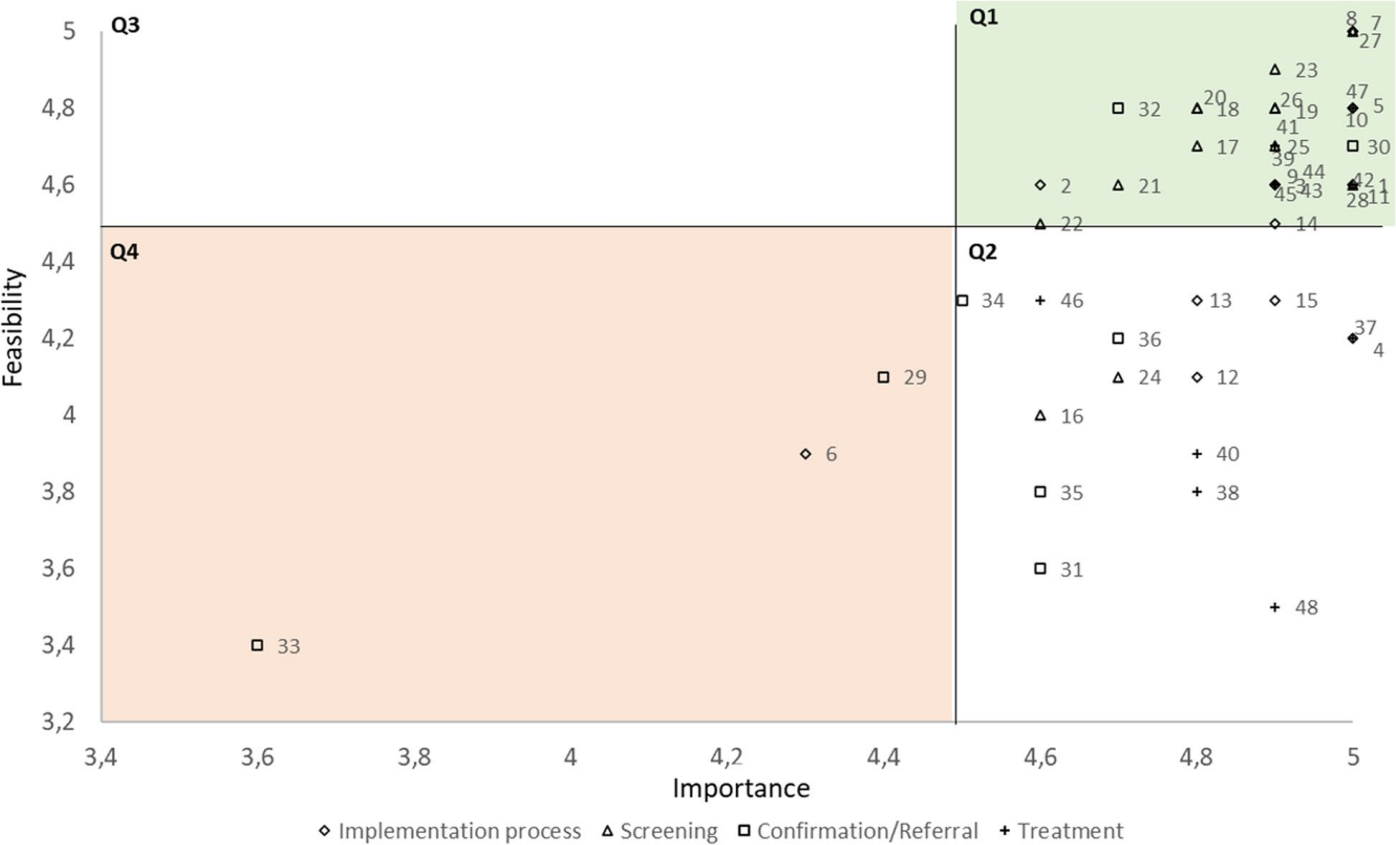
Go-zone graph plotting mean importance and feasibility scores for all 48 strategies based on stakeholders’ ratings

The strategies identified and discussed through the workshop were related to: develop stakeholder interrelationships (*N* = 8 individual strategies), train and educate stakeholders (*N* = 2), use evaluation and iterative strategies (*N* = 4, three for monitoring), utilize financial strategies (*N* = 1) (Table 1). Most strategies (67%) were in the go-zone (i.e. high importance, high feasibility) (Fig. 2) considered important, while concerns were raised about the feasibility of four strategies (27%) included in three different ERIC clusters (details below). One strategy (i.e. create partnerships) was considered to be of low importance and feasibility.

The workshop participants also suggested two additional strategies: 1) the creation of a national working group to collaborate on the implementation plan involving different stakeholders (National AIDS Council, the AIDS Control Division and the Mental Health Division) (ERIC strategy: development of stakeholder interrelationships); 2) the creation of a national directory of psychiatrists, where psychiatrists could indicate their availability every week to manage active suicidal risk (based on the same model as an existing Senegalese emergency medical service) (ERIC strategy: development of stakeholder interrelationships).

The intervention flow chart was retained as a strategy that needs to be adapted from one site to another (Table 1, Implementation strategy (IS) n°2). The choice of a mental health referent in each clinic was well received, but the importance of clearly defining their roles was reported (IS4). The workshop participants suggested working in pairs: a doctor and a social worker.

Regarding the creation of partnership with local institutions (IS6), the importance of the conditions for access to these training courses and the framework for implementing the therapy were reiterated. This strategy was discussed at length, but no clear procedure was established. Regarding the training of local mental health specialists (IS9), the workshop participants recommended to include psychologists and not only psychiatrists. The implementation plan was seen as critical (IS11) but the issue of project ownership was raised and the need to mobilize the different stakeholders at the very beginning was also reported. Maintaining continuous communication between the implementation planners and the adopters/implementers was reported as very important but with some concerns about feasibility, especially for communication costs (IS12). A recommendation for financial strategies (IS15) was to integrate these implementation strategies in other national and pre-existing strategies, either within the mental health or the HIV strategic plans. Some workshop participants also reported the need to prepare a conceptual note in which a cost hypothesis is formulated for each action and a specific funder is mobilized. With regard to monitoring meetings (IS13), their frequency was revised to every three months, in line with the rhythm of the existing technical health committees that already meet at this frequency.

### Screening step

The strategies identified and discussed through the workshop were related to: engage consumers (*N* = 1 individual strategies), train and educate stakeholders (*N* = 6, one for monitoring), change infrastructure (*N* = 4, one for monitoring), support clinicians (*N* = 1), provide interactive assistance (*N* = 1 for monitoring) (Table 1). The majority of the strategies were considered as important and feasible (85%) but two (15%) required adaptations (i.e. educate patients and use of a digital screening tool). One strategy was considered as part of another one (i.e. refresher training).

The need to educate patients about mental health and depression (IS16) was highlighted as important, but feasibility was discussed. The implementation team reported the development of educational videos that would be available through a QR code and/or displayed locally in clinics (with TV in the waiting room or in general health awareness actions). However, for some workshop participants, access will not be as easy for some patients depending on their particular situation and where they receive care. Thinking about different support was retained as an important need. Existing e-learning platforms for patients could also be a way forward. The workshop participants also insisted on the importance of training providers specifically on depression and HIV infection, but also including more information beyond this context to prepare professionals for other cases (IS17 & 19). Dose was discussed and adapted such that training would occur twice a year. The trained physician in charge could do the training for new providers but a trainer's manual was identified as a need to train new staff and organize supervision of activities. Newly trained physicians will be new trainers. Stakeholders agreed with the fact that to be inclusive of all patient profiles, language needs to be ethnically appropriate and adapted (IS18). The issue of access to health care for people who are deaf and hard of hearing was also raised and solutions were discussed: closer links should be established with the National Federation of People with Disabilities, particularly as there is a representative in each region. The creation of a memo sheet on symptoms of depression was well received and considered useful (IS20). The workshop participants noted the need to adapt the screening tool and to produce an administration manual to facilitate its use (IS23). Some concerns were raised about the digital tool, depending on the clinic. In some cases, clinics may have unreliable internet; in others, there may be insufficient materials for using a paper version. Role independence (the screener does not confirm or process) and confidentiality are the two elements that were considered highly important for this stage (IS27&25). The workshop participants agreed that the screener should be the provider with more time and who is closer to the patients. The choice will be adapted depending on the clinic profile. Pharmacists or community health workers could do this, but some members of the workshop participants insisted on the need to have a private space to do the screening and to keep the duration of the screening as short as possible. The use of a register, particularly in digital form, to monitor the screening step was well received (IS26). The version of the register presented during the workshop needed some clarification and adaptation, in particular to facilitate its integration into the national health monitoring forms. The new version of the register will be validated with the workshop participants when the protocol of the pilot study is written. This register could be completed by a social worker or a data manager on site, depending on clinic profile. Action dose was discussed and it was determined that the monitoring of this register could be organized once a month by the mental health referent. This register could be used to produce a monthly or quarterly report. The workshop participants also discussed the possibility of including this monitoring in the activities of regional health referral centers, which will need to include a referral psychiatrist. Refresher training for screening was not retained as a key strategy (IS22). The workshop participants felt that this was already included in the supervision step. The frequency of screening supervision (IS28) was discussed as necessary in the first week, then at week 3, month 3 and month 6.

### Diagnosis confirmation and case referral step

The strategies identified and discussed through the workshop were related to: engage consumers (*N* = 3 individual strategies), train and educate stakeholders (*N* = 2, one for monitoring), change infrastructure (*N* = 2), provide interactive assistance (*N* = 1 for monitoring) (Table 1). Two strategies were in the go-zone (25%), two other strategies were not considered as relevant (25%) (i.e. refresher training and bring patients with positive screening directly to physician in charge…). The second was largely discussed to be reformulated.

The strategy of taking a patient with a positive screening result, to the doctor in charge was considered less important and less feasible (I29) but the workshop participants agreed on the importance of confirming the diagnosis of depression on the same day as the depression screening. The strategy was reformulated this way (Table 3). The workshop participants discussed the need to give the patient a form indicating the need to confirm the diagnosis of depression and then he/she will follow the general clinical pathway to avoid stigmatization. For specific cases (i.e. patients collecting their medication without a scheduled consultation), priority access to consultation would be given. For patients diagnosed with depression, it was discussed to hold the pre-group session on the same day as diagnosis confirmation (IS31): the patient will go to the social worker’s office on his/her own and the pre-group session will be organized on the same day or during the week following the confirmation, depending on the availability of both the patient and group IPT trained staff. During confirmation, the use of adapted, simple and clear language was raised (IS32). A special focus on communicating the diagnosis of depression will be included in the training of doctors, including practical cases. For the decision tree (IS34), workshop participants recommended that its use be included in the training to explain well how it works. As for a digital diagnostic tool (IS35), some concerns were reported regarding the use of the WHO Mental Health Gap Action Programme (mhGAP) application tool (i.e. an WHO initiative to improve mental health care in LMICs) for the same reasons as described above in the screening process. As for screening, refresher training for confirmation (IS33) was considered an irrelevant strategy as it already was included in formative supervision sessions. The framework for supervision (IS36) needs to be well defined to avoid overwhelming the psychiatrist with requests. For example, it was discussed that telephone calls should be reserved for the management of patients at risk of suicide and the confirmation process should only be discussed during supervision sessions. Workshop participants agreed that supervision could be more easily carried out online, in groups and at regular intervals: after 1 month of formal depression care integration, 3 months after and every 6 months. Due to doctors’ turnover, whenever a doctor is set to leave, a formal training must be immediately arranged on site between the departing and incoming doctor.

### Treatment step

The strategies identified and discussed through the workshop were related to: engage consumers (*N* = 2 individual strategies), train and educate stakeholders (*N* = 3, one for monitoring), change infrastructure (*N* = 3, one for monitoring), support clinicians (*N* = 2), provide interactive assistance (*N* = 1 for monitoring), utilize financial strategies (*N* = 1) (Table 1). The majority were in the go-zone (58%). But, for four strategies (42%) included in three ERIC clusters, even importance was high, concerns were raised about the feasibility.

Calling patients the day before group IPT sessions was discussed (IS37), especially with regard to cost of calls. However, it was considered important to remind patients of group sessions to promote therapy adherence. The use of WhatsApp messages or eliciting the help from a patient in an IPT group to remind their follow group members of their upcoming therapy session might be viable solutions. Concerning the use of Interpersonal Counseling (IS38) (a brief, individual adaptation of Interpersonal Therapy) [50], especially for patients who could not come to group sessions, some workshop participants expressed strong concerns about this strategy, especially for fear of the difficulties to effectively oversee practices properly. The importance of training and supervision but also the framework for implementing this support were raised. Finally, the workshop participants agreed that alternative treatments could be proposed for very specific cases (i.e. workers, key population), but only when presenting symptoms are moderate. Moderate-to-severe or severe cases will need to be referred to a psychiatrist whenever participation in group IPT is not feasible or recommended. The training of two providers per clinic on group IPT was deemed necessary (IS45). Depending on staff availability per clinic, it was proposed that social workers, community health workers or peers could be trained. Owing to the necessity to reorganize working hours, this strategy was tepidly received (IS46). In each clinic, staff are under the supervision of different persons. Another complication comes from the fact that some supervisors are at times based outside of the clinic. This strategy and related action have been reformulated and will need to be adapted on a case-by-case basis. The source of funding for transportation costs was well received (IS48). There was support for the suggestion that the specific reimbursement amount be adapted to each patient based on distance traveled to and from treatment site. Nonetheless, some concerns about feasibility remained.

### Final implementation plan

In the final implementation plan, we included a total of 46 different implementation strategies (Tables 2 and 3). Our strategies were based on 21 different ERIC strategies and 8 different ERIC clusters. The three most represented clusters were "train and educate stakeholders" (12 selected strategies), "change infrastructure" (9 selected strategies) and "develop stakeholder interrelationships (8 selected strategies).

### Implementation protocol, materials and evaluation

**Fig. 3:**
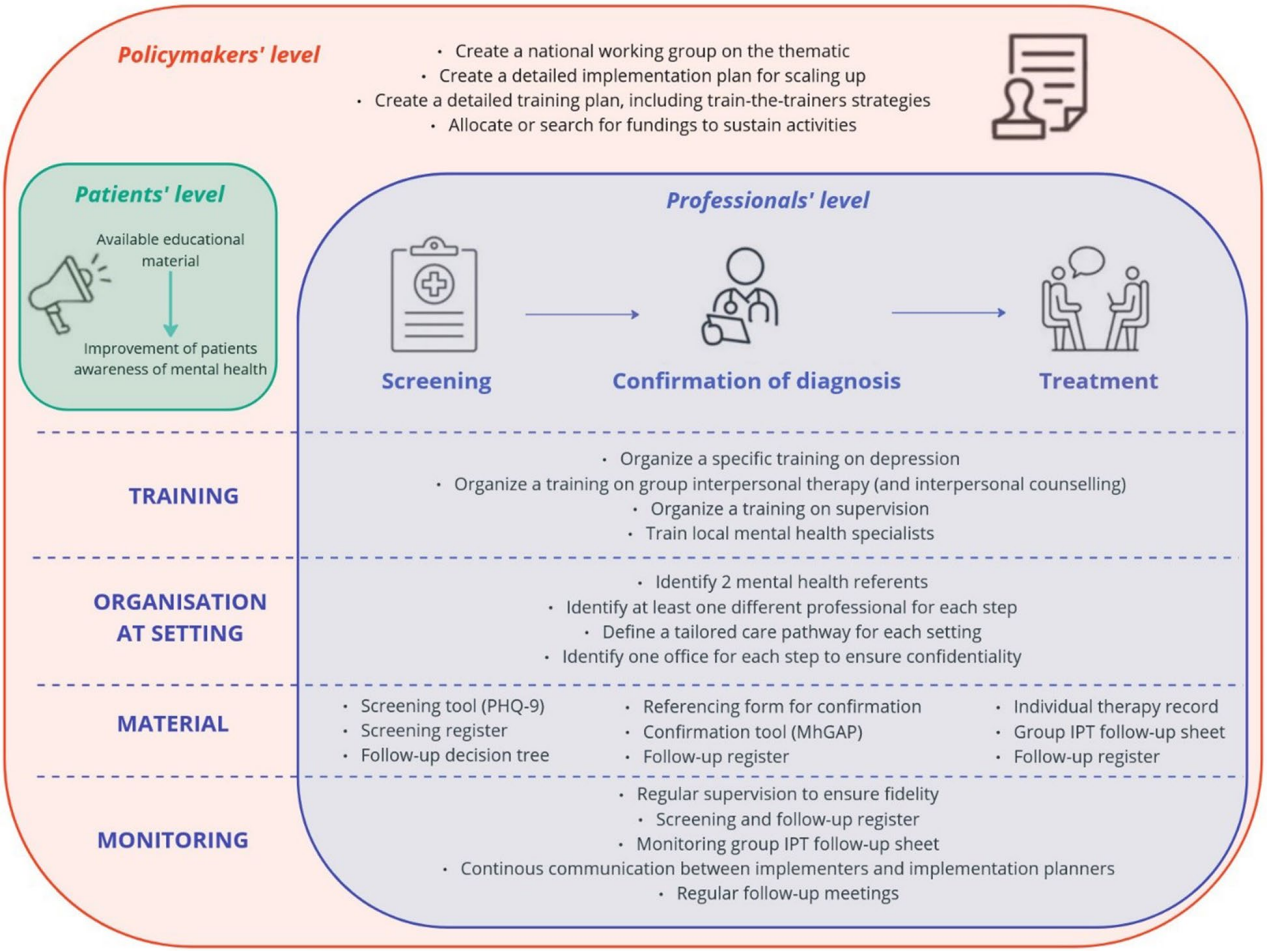
Summary of the implementation plan for integrating depression management into HIV care services in Senegal

A summary of the implementation plan for integrating depression management into HIV care services in Senegal is presented in Fig. 3. A detailed implementation protocol will be developed following the creation of the national working group. A training plan, including a train-the-trainers strategy, is required. Materials needed for implementation and monitoring have already been prepared. Some materials were used during our first experience of group IPT practice (i.e. individual therapy record, group IPT follow-up sheet) and approved by implementers in their initial version while others were discussed during the workshop (i.e. organization chart, screening and follow-up register, referral form for confirmation). A digital screening tool and mental health awareness tools will need to be developed. This implementation plan will be evaluated during a pilot study in Senegal in early 2025.

## Discussion

This report presents how we selected and co-designed implementation strategies for integrating depression management into HIV services in Senegal, involving stakeholders, in a context where limited resources, stigma, and mental health infrastructure are major challenges. Using Implementation Mapping, we identified and tailored strategies to address specific needs at different levels (i.e. individual, organizational and systemic) and developed the broad outline of an implementation plan.

The lack of a systematic approach to reporting the implementation of interventions, as observed in LMICs [35], and the lack of information about the process of planning and implementing evidence-based interventions [49, 51, 52] has been reported previously. Without a systematic approach, the transfer of research findings into everyday practice could be compromised, reducing the chances of evidence-based interventions being adopted, implemented and sustained in real-life conditions [53]. Implementation Mapping allows to present in detail the process facilitating the replication and improvement of intervention delivery, an important addition to the literature [35, 36, 54]. Implementation Mapping also allows the formulation of tailored and contextualized strategies to facilitate implementation and anticipate barriers to scale-up. In our work, we incorporated the practical experience of implementers who shared barriers and facilitators encountered during the delivery of group interpersonal therapy. During the workshop, implementers shared their perceptions, feelings, and experiences of group IPT in their respective services. The discussions allowed us to identify strategies and related actions that needed to be reformulated or adapted depending on the specific context and particularities of each setting (i.e. use of digital tool depending on internet access). The use of digital tools is becoming more widespread in health systems [55] but it should be adapted to the local context as well; the digital divide is significant in certain parts of the country and tailored solutions need to be provided in these settings. During the workshop, we were able to look back on the activities carried out as close as possible to the reality on the ground, to pool together the experiences of the different stakeholders and to look ahead to scaling up.

The use of brainstorming sessions with different stakeholders at the same time enabled them to compare and contrast their sometimes-divergent opinions, and to bring out new perspectives and potential barriers to be overcome. Firstly, the need for a detailed training plan appeared to be extremely important, particularly due to the costs of training and the strain and shortage of local psychiatrists to supervise activities and manage suicide risk when needed. The use of a train-the-trainers strategy was particularly important to address these challenges [51–53]. Secondly, the representatives of psychiatry who participated in the workshop expressed their reluctance to provide training in interpersonal counselling (individual support) and to establish partnerships with certain national training institutions. Their concerns were linked to the fact that training requires a precise framework and regular supervision by a mental health specialist, especially for non-specialists. Mental health task shifting is relatively new in Senegal [3]. This change requires not only standardization of training practices, but also an evolution of clinical practices and power dynamics, which can be a challenging process. It requires standardization of training practices but also changes in clinical practices and power dynamic. Thirdly, concerns were raised about inclusive access to healthcare, particularly for people who are deaf and hard of hearing. This will require the establishment of a specific partnership with local associations working in this field. Fourthly, the creation of a national working group to look at scaling up and incorporating strategies into national HIV guidelines was unanimously approved, reflecting the desire to build a sustainable project and ensure the long-term future of the program.

In our implementation plan, we also decided to include strategies to educate patients about mental health and depression to fight for stigmatization and encourage them to seek help when needed. Additionally, we adapted the care pathway to take account of patients' need for confidentiality and to limit stigmatization in clinics. One study, conducted not in SSA but in the United States, also highlighted the need to understand the patient as a whole and to adapt to their experience of the illness in order to ensure the success of the service [56].

Moreover, workshop participants indicated the importance of including monitoring strategies to ensure fidelity but also highlighted the need to iteratively adjust the identified strategies based on the clinic’s current resources. Incorporating different monitoring methods (registers, ongoing exchanges between planners and implementers, etc.) will help to implement strategies and identify obstacles. Monitoring will also allow to deal with the positive or negative consequences that will emerge from the implementation of additional measures in the care structure [43, 57].

In addition to the use of Implementation Mapping, the main strengths of this work were the integration of the patients’ perspective on each step of depression care (i.e. perceptions, facilitators and barriers reported in previous works) and the in-depth discussions on strategies with various stakeholders with field experience. However, some limitations could be reported. Firstly, differences in consistencies between voting on the strategy formulation, importance and feasibility of certain strategies can complicate interpretation. But it is important to report this sometimes-divergent information to highlight the discussions that took place during the workshop. These discussions revealed different stakeholders' perspectives on some strategies, particularly those related to training, supervision and time reorganization. Sometimes no clear procedure was established to describe in detail the action related to the selected strategy (i.e. creation of partnership with local institutions), highlighting the difficulties in the creation of new processes and the need to structured approach. Secondly, we did not have any stakeholders participating in the workshop representing care services in rural or remote areas, but we were working with professionals from four different clinics, representing different care contexts in Senegal, and some of the professionals had knowledge of the rural/remote context from previous work in those regions. We also recognize that these strategies could not cover all levels of care in Senegal. A minimum number of professionals is needed to provide the different levels of care (screening, confirmation, treatment) independently, in particular to cope with the workload, and thus these strategies will likely not apply to the two lowest levels of the health pyramid in Senegal (health posts and "cases de santé"). In the general process step, we included specific strategies to work on a detailed implementation and training plan but also to define a budget allocated to this dissemination and identify resources. Adaptation to rural settings will be a part of those discussions, as we know some adaptations will be required. Thirdly, while participants have an history of working together leading to open discussions, we could not exclude the possibility that power imbalances among participants subtly influenced workshop dynamics and strategy prioritization. Fourthly, even though patients’ perspectives had been explored in our previous works to understand their perceptions and needs regarding depression treatment, informing the selection of strategies, they were not involved in the workshop owing to logistical limitations (i.e., patients may not be open to expressing opinions in workshops with providers and policymakers, and we did not have the capacity to conduct parallel workshops). Their participation would be important for future phases of co-design.

The implementation protocol related to this work, is currently being written in detail in collaboration with the National AIDS Council, the AIDS Control Division and the Mental Health Division at the Ministry of Health and Social Action. In the second quarter of 2025, we will initiate a pilot project to evaluate selected strategies in clinics where group IPT has already been used. We will use a mixed-methods study design to document the impact of the optimized selected strategies on the routine integration of depression management in these clinics. In particular, we will assess the impact of new depression education materials on the attitudes of PWH towards screening, the experience of implementing systematic depression screening and the perceptions of the tailored care pathway. This project will allow us to learn from the implementation of these optimized strategies before scaling up group IPT across Senegalese HIV services.

## Conclusion

Integrating depression management into HIV care requires appropriate solutions in contexts where resources are limited. Early involvement of stakeholders is an important asset in designing implementation strategies that are in line with available resources and training. This study, conducted in Senegal, has defined an implementation plan that identifies key strategies and their specifications for each care step, by integrating the experiences of different stakeholders. This approach provides a blueprint for integrating depression management into HIV services across the country and could serve as a guide for developing similar programs in other low-resource contexts.

## Supplementary Information

Supplementary Material 1.

## Data Availability

All data relevant to the study are included in the article or uploaded as supplementary information.
