# Supplementary material for: Stakeholder engagement to co-design implementation strategies for integrating depression management into HIV care services in Senegal
Source: Implement Sci Commun. 2025 Nov 17;6:123. doi: 10.1186/s43058-025-00801-1 (PMC12625524; doi:10.1186/s43058-025-00801-1)
Supplement: Supplementary file 1 — Supplementary Material 1. [file 43058_2025_801_MOESM1_ESM.docx]

**Supplemental Material**

**
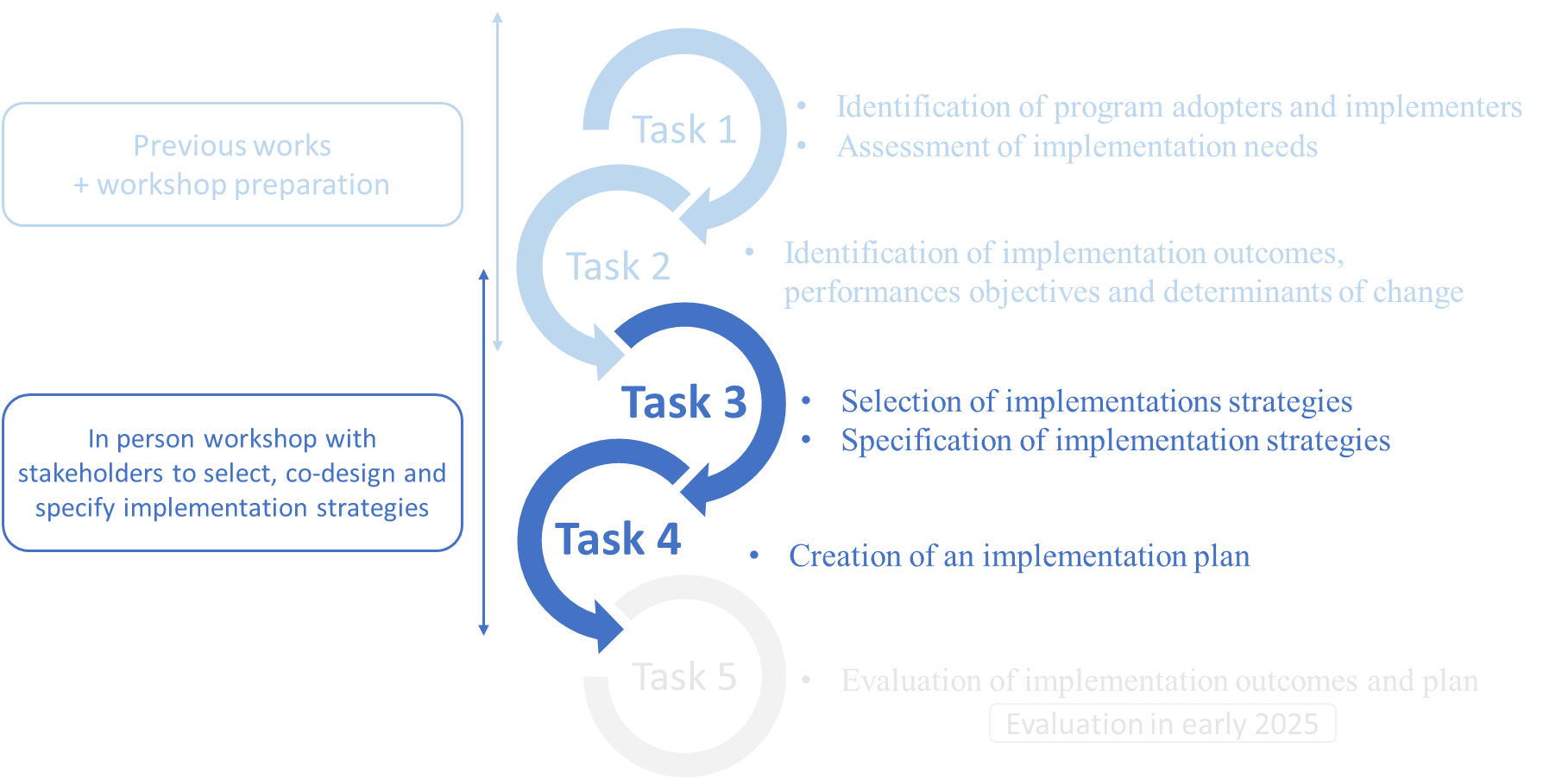
**

**Figure S1:** Flow Diagram of Implementation Mapping Approach used in our work

**
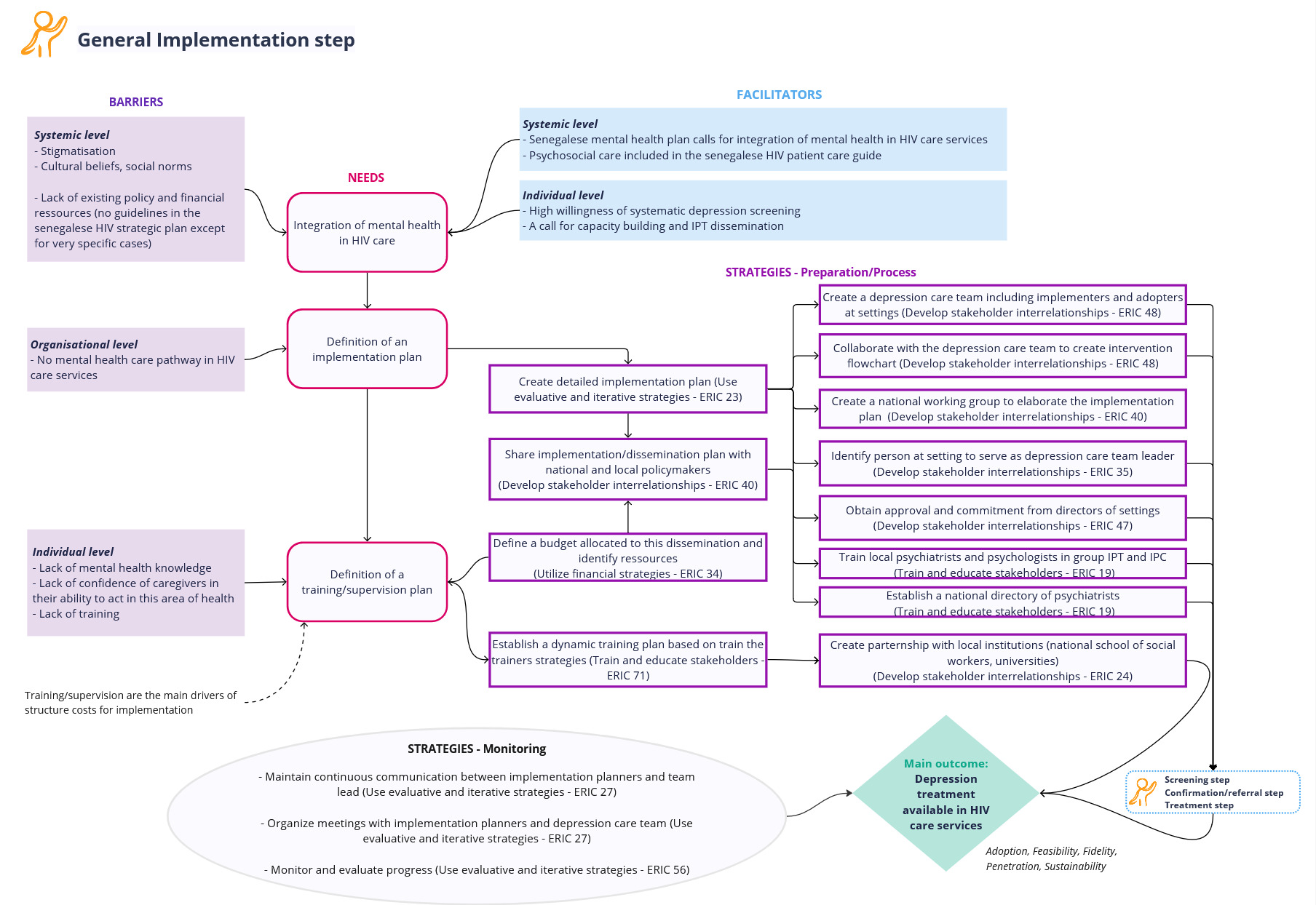
**

**Figure S2:** Logic model – General Implementation Process


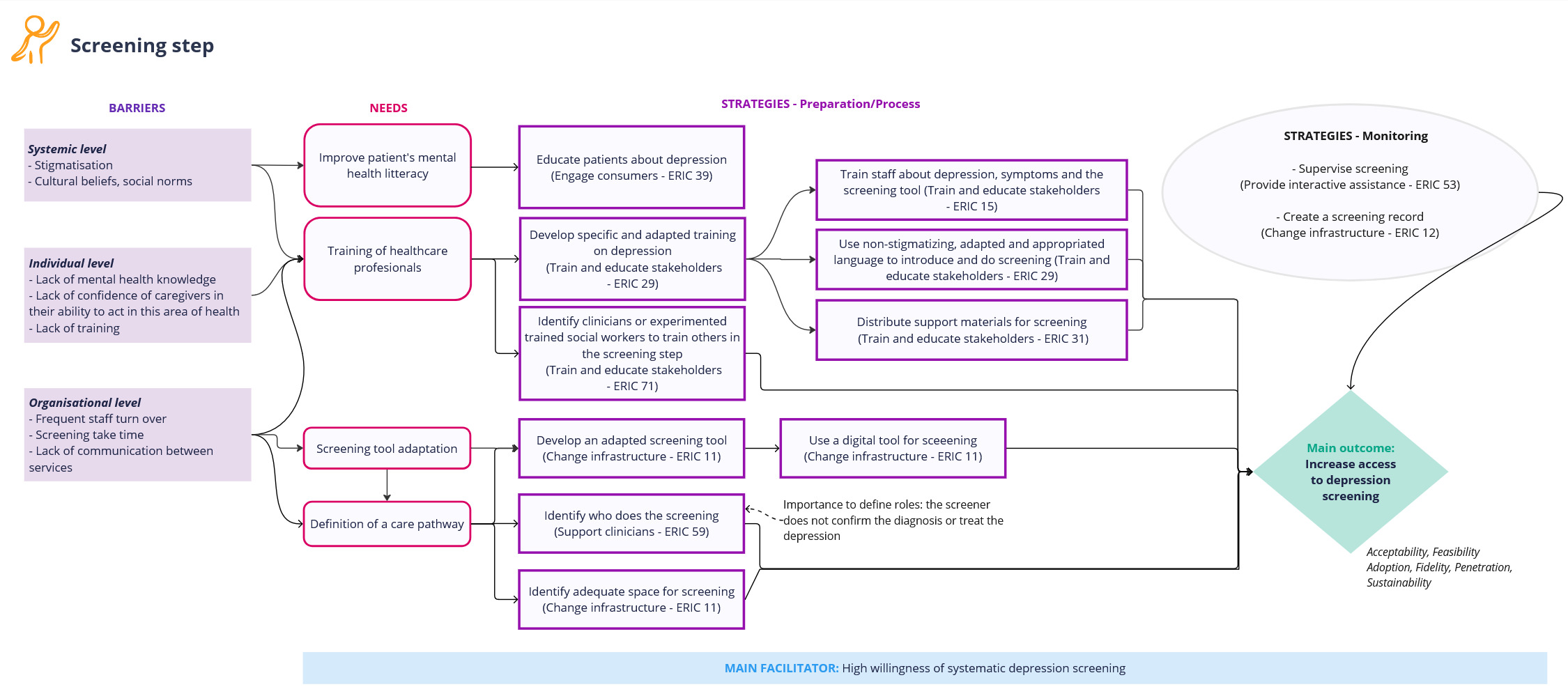


**Figure S3:** Logic model – Screening Step

**Figure S3:** Logic model – Confirmation/Referral Step


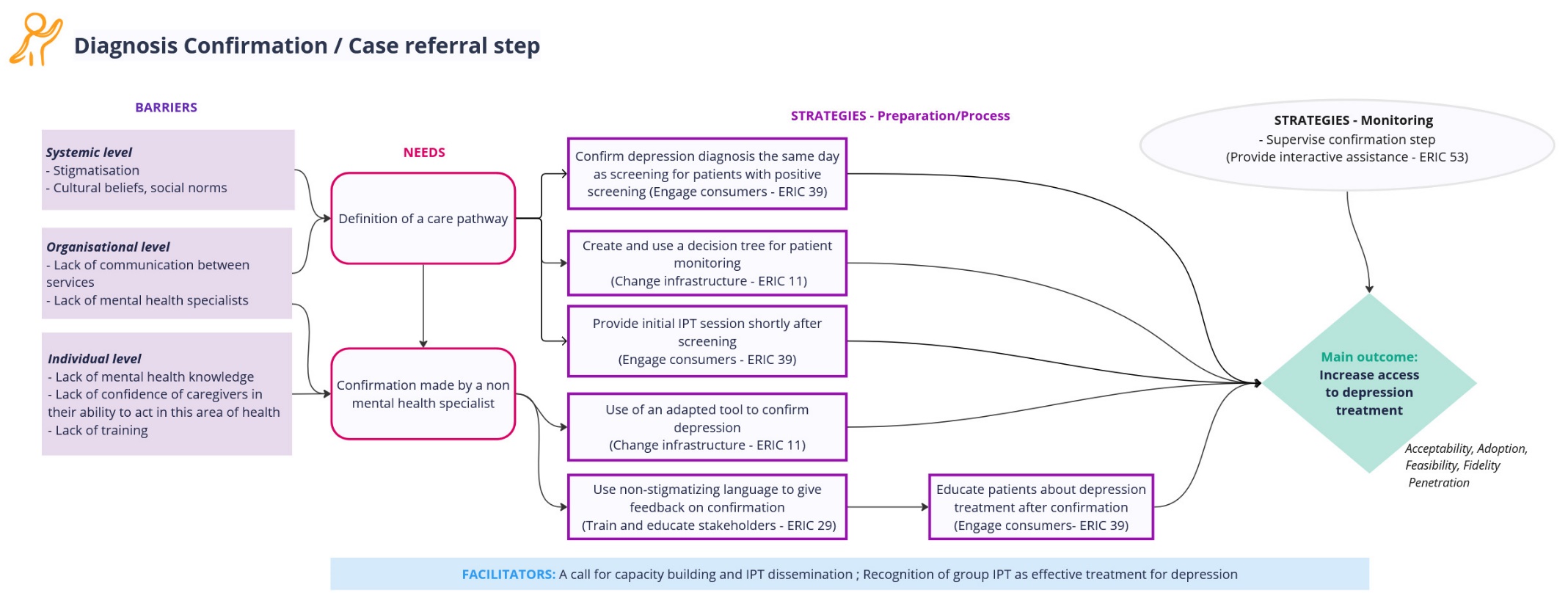


**Figure S4:** Logic model – Diagnosis confirmation / case referral Step


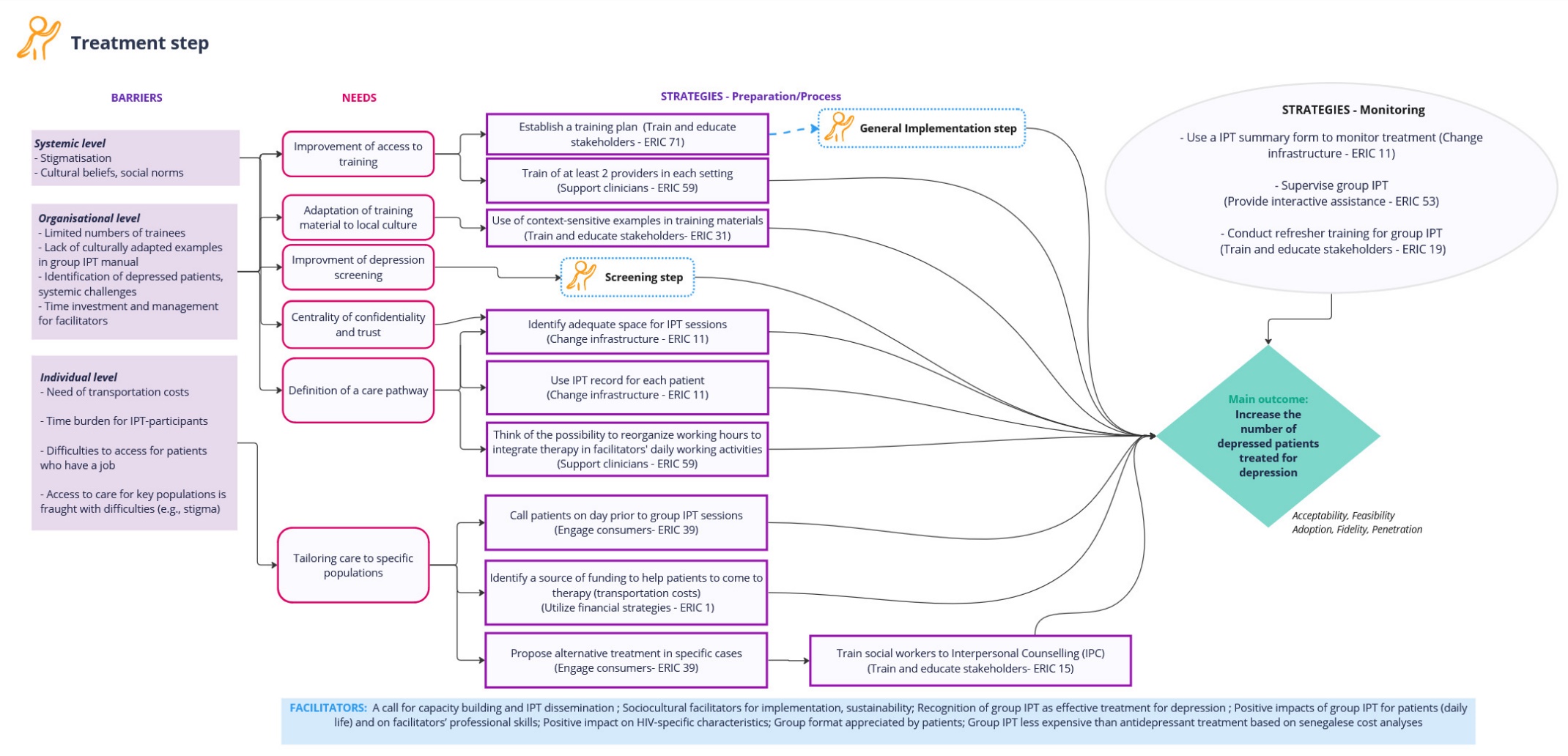


**Figure S5:** Logic model – Treatment Step

**Table S1:** Implementation outcomes and performance objectives for integrating depression management into HIV care services in Senegal

|  |  | **Measures** | | | **Performance objectives** |
| --- | --- | --- | --- | --- | --- |
|  |  | **Screening step** | **Referral step** | **Treatment step** |  |
| **Acceptability*** | Staff | Acceptability of doing screening Acceptability of digital screening tool | Acceptability of doing confirmation by physician (i.e non mental health specialist) | Acceptability of doing group IPT | Steps acceptables for providers |
|  | Patient | Acceptability of awareness information Refusal rate to be screened | Refusal rate to be confirmed | Drop-out rate in group IPT | 90% accept to visualize awareness materials 90% of patients accept to be screened 90% with positive screening accept to be referred 90% patients with a diagnosis of depression accept group IPT as a treatment |
|  |  |  | Refusal rate to participate in group IPT | Satisfaction (treatment) |  |
| **Adoption*** | Setting | % providers (involved in the care pathway) screening, referring & delivering group IPT | | | 90% for each step |
|  |  | % setting exclusion, % settings approached that participate + Characteristics of these settings + for staff | | |  |
|  |  | Climate of implementation | | |  |
| **Feasibility* (including retention)** | Staff | Time for screening + Workload => evaluation of systematic screening | Workload | Workload | Wait time before treatment start <3 weeks 80% of retention in group IPT >90% attendance High management of suicidal risk 90% of patients satisfied by group IPT |
|  | Patient |  | Wait time before treatment start | Retention: % group IPT sessions completed + Level of attendance + % justified nonattendance |  |
|  |  |  |  | Death per suicide or hospitalization for imminent risk for suicide |  |
|  |  |  |  | Evolution of suicidal ideations |  |
|  |  |  |  | Antidepressant prescriptions |  |
|  |  |  |  | Therapy recommendation to other patients |  |
| **Fidelity** | Staff | % correctly completed screening | % correctly completed referrals |  | 95% correctly completed screening 95% correctly completed referrals 95% correctly facilitated group IPT |
|  |  |  |  | Group IPT fidelity checklist; Supervision group IPT fidelity checklist |  |
|  |  |  |  |  |  |
| **Penetration** | Patient | % patients screened | % patients who access to referral when necessary | % referred patients who have group IPT | 90% of patients are screened  90% of patients with a positive screening access to confirmation  90% of patients with positive diagnosis are treated |
|  |  |  |  |  |  |
| **Sustainability*** | Staff | Post evaluation penetration and retention | | | Indicators are maintained in the sustainable phase |
|  |  | Maintenance of the program at >=6 months | | |  |
|  |  | Long-term Adaptation (which elements retained AFTER program completed) | | |  |
|  |  | Workload | | |  |
|  | ****Qualitative data in supplement*** | |  |  |  |
